# A post-pandemic snapshot of the magnitude of COVID-19 in Brazil: A countrywide study

**DOI:** 10.1016/j.bjid.2024.104496

**Published:** 2024-12-21

**Authors:** Eduardo Ribes Kohn, Maristela Bohlke, Antônia Almeida, Leandro Janelli, Luciana Monteiro Vasconcelos Sardinha, Fernando C. Wehrmeister, Pedro Curi Hallal

**Affiliations:** a Universidade Federal de Pelotas, Programa de Pós-Graduação em Educação Física, Pelotas, RS, Brasil; b Universidade Católica de Pelotas, Programa de Pós-Graduação em Saúde, Pelotas, RS, Brasil; c Vital Strategies, São Paulo, SP, Brasil; d Universidade Federal de Pelotas, Programa de Pós-Graduação em Epidemiologia, Pelotas, RS, Brasil; e University of Illinois Urbana-Champaign, Department of Kinesiology and Community Health, Urbana, IL, USA

**Keywords:** COVID-19, Long COVID, Adults, Brazil

## Abstract

**Objective:**

To outline the features of COVID-19 in Brazil through a countrywide telephone survey.

**Methods:**

Data from the Telephone Survey of Risk Factors for Chronic Noncommunicable Diseases During the Pandemic (Covitel), a telephone survey of individuals aged 18 years or older from all macro-regions of Brazil, were used. The questionnaire included sociodemographic characteristics and outcomes related to COVID-19 infection, severity, vaccination, and use of masks.

**Results:**

Data revealed that 34.7 % (95 %CI 32.4 – 37.1) of the population had been diagnosed with COVID-19, and 10.1 % (95 %CI 7.9 – 12.7) of those required hospital admission. The prevalence of COVID-19 diagnosis increased with education level: <8 years (26.6 % [95 %CI 23.1 – 30.7]), 9–11 years (33.4 % [95 %CI 29.4 – 37.7]), and >11 years (53.2 % [95 % CI 49.7 – 56.8]). Nevertheless, the hospitalization rate of Brazilians with more than eleven years of education was lower (5.8 % [95 %CI 4.3 – 7.6]). In 2023, 92.9 % (95 %CI 90.9 – 94.4) of the Brazilian population was fully vaccinated against COVID-19, but only 37.2 % (95 %CI 33.5 – 40.9) have received the updated vaccinal scheme (two doses and two boosters). During the pandemic outbreak, 81.9 % (95 %CI 79.4 – 84.2) reported always using face masks. However, only 16.1 % (95 %CI 13.5 – 19.0) maintained this practice in 2023.

**Conclusion:**

There were inequalities in COVID-19 testing in Brazil. Testing and vaccination policies implemented in the COVID-19 pandemic must be reevaluated by the Brazilian government.

## Introduction

In December 2019, China reported the first cases of the disease associated with the novel coronavirus SARS-CoV-2 to the World Health Organization (WHO), precipitating a global health crisis of unprecedented scale.^1^ Within a month, COVID-19 had already infected more than 4500 people. In February 2020, the first case of the disease was reported in Brazil, a country that came to be the Latin American epicenter of the pandemic.^2^

Since the beginning of the pandemic, the WHO has recommended mass testing as the main strategy to contain the virus, for which no vaccine was available at the time.^3^ However, due to strategic, political, and economic difficulties, Brazil took longer to make the most accurate tests available.^4^ In December 2020, data on the effectiveness of newly developed vaccines were released and approved for emergency use in occidental countries.^5^ The first vaccine in Brazil was applied in January 2021.^6^ At the end of 2021, after research found that vaccine effectiveness wanes over time, Brazil started administering booster vaccine doses.^7^

After the end of the pandemic on May 5, 2023, the virus has killed up to 7 million people, around 700,000 of them in Brazil. A subset of patients who survived COVID-19 have developed a range of clinical symptoms that persisted after the acute phase, including dyspnea, fatigue, headache, and loss of smell and taste. This condition has received many labels, such as post-acute COVID-19 syndrome, persistent post-COVID-19 syndrome, and long-COVID-19.^8^

Brazil faced unique challenges exacerbated by socioeconomic factors that contributed to the rapid increase in COVID-19 cases during the initial course of the epidemic.^4^ Understanding the complex challenges presented by the COVID-19 pandemic, which has significantly affected human life for more than three years, and the implications of the long COVID-19 is crucial for healthcare planning in the future. This study aims to outline the features of COVID-19 and its lasting effects in Brazil through a telephone survey that represents the entire population.

## Methods

Data from the *Telephone Survey of Risk Factors for Chronic Noncommunicable Diseases During the Pandemic* (Covitel), a telephone survey of individuals aged 18 years or older from all macro-regions of Brazil, were used. The Covitel was conducted by Vital Strategy and Federal University of Pelotas (UFPel). In this survey, a specialized research company interviewed people in the first quarter of 2023. The Covitel 2023 project was approved by the Higher School of Physical Education Research Ethics Committee, Federal University of Pelotas, Brazil (nº 5727,059). Methodological aspects were published by Hallal and collaborators.^9^

The Covitel calculated the sample size needed to estimate the risk factors for Chronic Noncommunicable Diseases (NCDs) in the Brazilian population with a 95 % Confidence Interval and a margin of error of three percentage points. The calculation estimated the need for 1800 interviews in each region of the country, with 900 interviews conducted via landline phones and 900 via mobile phones. They used Random Digit Dialing to generate lists of all eligible phones. We randomly selected lines, stratified by landline and mobile phone users, and proceeded to the interviews using the included phone numbers. Company phones and out-of-service lines that did not respond after six attempts at different times across all seven days of the week, including holidays, were considered ineligible.

The interviewer sorted household members aged 18 and over in ascending order for landline numbers and randomly selected the interviewee. When contacting mobile phone numbers, the owner of the device was interviewed. If necessary, it was scheduled another time for the interview. Before conducting the interview, the interviewer read and asked for agreement to the Informed Consent Form. The interview was guided by a computer system, and responses were recorded electronically. This interview system allowed for daily auditing of 10 % of the sample with real-time monitoring of the interviewer's screen.

The questionnaire included sociodemographic characteristics and outcomes related to COVID-19 infection, severity, vaccination, and use of masks. Sociodemographic characteristics were a) Sex (female and male); b) Race/color (white, black, brown, yellow, and indigenous); c) Country region (South, Southeast, Midwest, North, and Northeast); and d) Education level (elementary school, middle school, high school, degree, graduate school and never studied). We analyzed eight outcomes related to the COVID-19 pandemic: a) COVID-19 diagnosis; b) Long COVID-19 symptoms; c) Frequency of COVID-19 diagnosis; d) Hospitalization due to COVID-19; e) COVID-19 vaccinations; f) Number of COVID-19 vaccine doses; g) Use of face masks during the COVID-19 pandemic; h) Face masks usage in 2023.

We conducted descriptive analyses to estimate the occurrence of the outcome according to sociodemographic factors. Complex data analysis was based on data from the 2010 Brazilian census carried out by the Brazilian Institute of Geography and Statistics (IBGE). The sample was stratified based on geographic region (Northeast, North, Southeast, South and Central-West), sex (male and female), age (18‒34; 35‒49 and 50-years or more) and education (0‒11-years and 12-years or more of schooling). Adaptations were made for age (the 18‒19 age group corresponds to 2/5 of the IBGE 15‒19 age category) and education (the 0‒11 years of study category was created for those with less than secondary education in the IBGE categories). We used Stata 18 for IOS to analyze the dataset (Stata Corp. College Station, TX, USA).

## Results

A total of 9038 individuals were interviewed. Most of the respondents (59.1 %) were female, 47.3 % were aged 50-years or older, and 48.3 % declared themselves as having black or brown skin color.

The findings revealed that 34.7 % (95 % CI 32.4–37.1) of the population had been diagnosed with COVID-19 by the end of the pandemic, 10.1 % (95 % CI 7.9–12.7) needing hospital admission. In 2023, 95.1 % (95 % CI 93.6–96.2) of the Brazilian population had received at least one dose of the COVID-19 vaccine, 92.9 % (95 % CI 90.9–94.4) were fully vaccinated (two doses), and 37.2 % (95 % CI 33.5–40.9) have received the updated vaccinal scheme (two doses and two boosters) at the survey time. 81.9 % (95 %CI 79.4–84.2) reported always using face masks during the pandemic outbreak, but only 16.1 % (95 % CI 13.5–19.0) maintained this practice in 2023. Memory loss was the most frequent long COVID-19 symptom, reported by 34.1 % (95 % CI 31.2–37.2) of the infected individuals.

The Midwest (42.1 % [95 % IC 35.9–48.7]) region population had significantly more prevalence of COVID-19 diagnoses than Northeast (31.0 % [95 % CI 26.9–35.6]). Midwest region population was less vaccinated with two boosters of COVID-19 vaccine (27.5 % [95 % CI 23.4–32.2]) than that of the Southeast (39.4 % [95 % CI 33.2–45.9] and Northeast (40.3 % [95 % CI 36.5–44.1]) regions. The use of face masks was similar among the Brazilian regions during the pandemic outbreak. In 2023, a smaller proportion of the population of the South region (8.4 % [95 % CI 6.2–11.3]) reported maintaining using always face masks (Table 1).

**Table 1:** Sample characteristics, diagnosis of COVID-19 infections in Brazil.

	Number of COVID-19 infections			
	None	One	Two	Three or more
	% (95 % CI)	% (95 % CI)	% (95 % CI)	% (95 % CI)
**Country region**				
Mid-West	57.9 (51.3 – 64.1)	42.1 (35.9 – 48.7)	13.4 (10.5 – 16.9)	3.5 (2.4 – 4.9)
Northeast	69.0 (64.5 – 73.3)	31.0 (26.9 – 35.6)	8.8 (6.7 – 11.5)	1.9 (1.0 – 3.5)
North	67.3 (61.8 – 72.4)	32.7 (27.7 – 38.6)	9.9 (8.4 – 11.6)	3.4 (2.7 – 4.2)
Southeast	65.0 (61.4 – 68.6)	35.0 (31.6 – 38.7)	8.7 (7.6 – 10.2)	1.6 (1.2 – 2.2)
South	62.6 (58.9 – 66.1)	37.4 (33.9 – 41.1)	10.3 (8.5 – 12.5)	2.5 (1.1 – 5.3)
**Sex**				
Male	66.9 (63.5 – 70.3)	33.1 (29.9 – 36.6)	8.2 (6.9 – 9.8)	1.7 (1.1 – 2.7)
Female	63.9 (61.5 – 66.4)	36.1 (33.8 – 38.7)	10.6 (9.1 – 12.1)	2.4 (1.8 – 3.3)
**Education**				
0–8 years	73.4 (69.4 – 77.1)	26.6 (23.1 – 30.7)	6.5 (4.9 – 8.5)	1.5 (0.7 – 3.1)
9–11 years	66.6 (62.3 – 70.6)	33.4 (29.4 – 37.7)	9.1 (7.3 – 11.3)	1.9 (1.1 – 3.3)
12+ years	46.8 (43.3 – 50.4)	53.2 (49.7 – 56.8)	16.1 (14.3 – 18.1)	3.7 (2.5 – 5.4)
**Skin color**				
White	61.8 (58.7 – 64.9)	38.2 (35.2 – 41.3)	10.2 (8.9 – 11.7)	2.3 (1.6 – 3.3)
Non-white	68.6 (65.5 – 71.6)	31.4 (28.4 – 34.6)	8.9 (7.4 – 10.8)	1.4 (1.4 – 2.7)

**Fig. 1:**
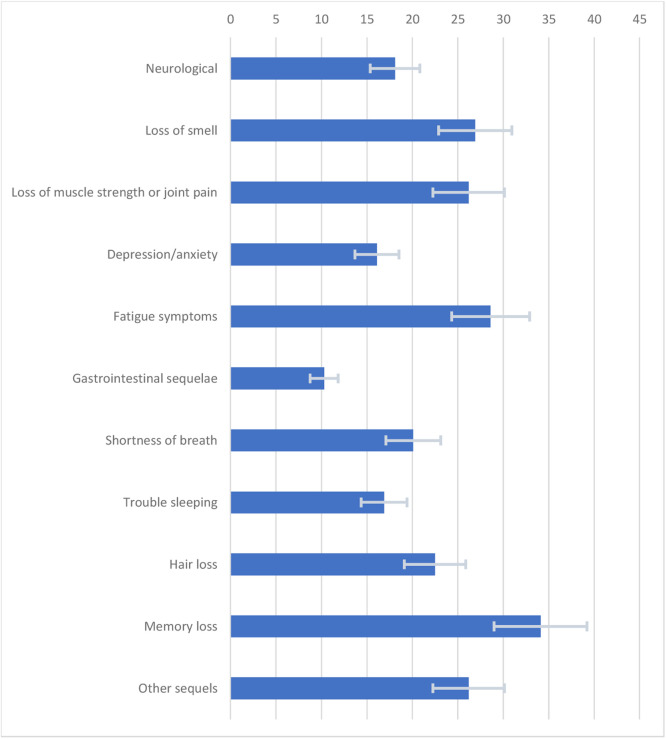
Sequelae after 6-months of COVID-19 diagnosis in Brazil.

The COVID-19 vaccination was different between male (94.0 % [95 % IC 91.6–95.8]) and female (97.4 % [95 % IC 96.5–98.1]) Brazilians. Females completed two boosters of COVID-19 vaccination (42.9 % [95 % CI 38.2–47.8]) more frequently than males (31.7 % [95 % CI 27.9–35.6]). Female Brazilians also used face masks more frequently during the pandemic outbreak (90.1 % [95 % CI 87.4–92.4]) and in 2023 (21.5 % [95 % CI 17.8–25.6]) than the male population (73.1 % [95 % CI 68.8–77.1] and 10.3 % [95 % CI 8.3–12.7], respectively). The hospitalization rate was not different between sexes (Fig. 1 and Table 2).

**Table 2:** Hospitalization due to COVID-19 infection and symptoms after 6-months of infection in Brazil.

	Hospitalized due COVID? (Among those with at least one infection)	Any symptom after 6-months of infection? (Among those with at least one infection)
	**% (95 % CI)**	**% (95 % CI)**
**Country region**		
Mid-West	10.9 (6.7 – 17.2)	42.1 (35.8 – 48.6)
Northeast	7.1 (4.5 – 11.4)	30.9 (26.7 – 35.5)
North	9.2 (6.1 – 13.4)	32.7 (27.6 – 38.2)
Southeast	11.4 (7.8 – 16.3)	34.9 (31.3 – 38.6)
South	10.7 (7.3 – 15.3)	37.4 (33.9 – 41.0)
**Sex**		
Male	10.6 (7.9 – 14.2)	33.0 (29.7 – 36.4)
Female	9.6 (6.9 – 13.2)	36.0 (33.6 – 38.5)
**Education**		
0–8 years	11.8 (8.5 – 16.2)	26.5 (22.8 – 30.6)
9–11 years	12.7 (8.6 – 18.7)	33.4 (29.4 – 37.7)
12+ years	5.8 (4.3 – 7.6)	53.2 (49.6 – 56.6)
**Skin color**		
White	11.3 (8.4 – 15.0)	38.2 (35.1 – 41.3)
Non-white	8.7 (6.3 – 11.9)	31.4 (28.4 – 34.6)

The prevalence of COVID-19 diagnosis increased according to the education level (< 8-years: 26.6 % [95 % CI 23.1–30.7]; 9–11-years: 33.4 % [95 % CI 29.4–37.7]; > 11-years: 53.2 % [95 % CI 49.7–56.8]). Nevertheless, the hospitalization rate of Brazilians with more than eleven years of education (5.8 % [95 % CI 4.3–7.6]) was lower than that of individuals with less than eight years (11.8 % [95 %CI 8.5–16.2]). The prevalence of COVID-19 vaccination was higher among individuals with more than 11-years of formal education (97.7 % [95 %IC 96.9–98.2]) than among those who had less than 8-years of education (93.8 % [95 % IC 91.3–95.6]). The percentage of two boosters of vaccination was lower among individuals with 9–11-years of education (29.2 % [95 % CI 24.7–34.1]). Reporting always mask use during the COVID-19 pandemic was not different between education groups. The frequency of the report of always using masks was higher among individuals with < 8-years of education in 2023 (21.8 % [95 % CI 17.3–27.1]) (Table 3).

**Table 3:** Number of COVID-19 vaccine shots taken in Brazil.

	Number of COVID-19 vaccine shots				
	None	One	Two	Three	Four
	% (95 % CI)	% (95 % CI)	% (95 % CI)	% (95 % CI)	% (95 % CI)
**Country region**					
Mid-West	6.7 (3.7 – 11.6)	93.3 (88.4 – 96.3)	89.4 (82.8 – 93.7)	65.6 (58.3 – 72.2)	27.5 (23.4 – 32.2)
Northeast	4.2 (2.4 – 7.1)	95.8 (92.9 – 97.6)	93.7 (90.2 – 95.9)	76.7 (69.8 – 82.4)	40.3 (36.5 – 44.1)
North	5.5 (3.9 – 7.5)	94.5 (92.5 – 96.3)	89.7 (85.4 – 92.9)	64.4 (56.1 – 71.9)	30.2 (24.9 – 36.2)
Southeast	3.6 (2.2 – 5.9)	96.4 (94.1 – 97.8)	95.2 (92.5 – 96.9)	76.2 (70.5 – 81.1)	39.4 (33.2 – 45.9)
South	4.2 (2.5 – 6.9)	95.8 (93.1 – 97.5)	92.5 (88.7 – 95.0)	69.6 (62.5 – 75.9)	35.3 (29.1 – 42.0)
**Sex**					
Male	5.9 (4.2 – 8.5)	94.0 (91.6 – 95.8)	91.6 (88.5 – 93.9)	68.6 (63.5 – 73.3)	31.7 (27.9 – 35.6)
Female	2.6 (1.9 – 3.5)	97.4 (96.5 – 98.1)	95.4 (93.9 – 96.6)	78.5 (74.4 – 82.1)	42.9 (38.2 – 47.8)
**Education**					
0‒8 years	6.2 (4.4 – 8.7)	93.8 (91.3 – 95.6)	91.9 (88.9 – 94.1)	75.1 (69.7 – 79.7)	42.2 (38.1 – 46.5)
9‒11 years	2.6 (1.5 – 4.7)	97.4 (95.3 – 98.5)	94.2 (91.6 – 95.9)	68.8 (64.3 – 73.0)	29.2 (24.7 – 34.1)
12+ years	2.3 (1.8 – 3.1)	97.7 (96.9 – 98.2)	96.2 (94.9 – 97.1)	77.9 (74.2 – 81.3)	39.6 (35.3 – 43.9)
**Skin color**					
White	3.2 (1.8 – 5.6)	96.8 (94.4 – 98.2)	94.8 (91.6 – 96.9)	75.2 (70.2 – 79.5)	39.9 (35.7 – 44.3)
Non-white	4.7 (3.3 – 6.7)	95.3 (93.3 – 96.7)	92.9 (90.6 – 94.7)	72.8 (68.4 – 76.8)	35.2 (31.8 – 38.8)

Brazilians with non-white skin color (31.4 % [95 % CI 28.4–34.6]) presented a lower frequency of COVID-19 diagnosis than white people (38.2 % [95 % CI 35.2–41.3]). However, the hospitalization rate was similar among the ethnic groups. The updated COVID-19 vaccination, vaccine doses, and mask use was also similar between educational groups (Table 4)

**Table 4:** Mask using during pandemic and in 2023, according to selected variables in Brazil.

		Mask use				
		Never	Sometimes	Often	Always	Don't know
Variable	When?	% (95 % CI)	% (95 % CI)	% (95 % CI)	% (95 % CI)	% (95 % CI)
Country region						
Mid-West	During pandemic	1.7 (0.8 – 3.4)	5.7 (4.3 – 7.7)	9.2 (7.3 – 11.8)	82.9 (80.8 – 84.8)	0.3 (0.2 – 1.0)
	In 2023	43.6 (37.6 – 49.7)	31.1 (25.9 – 36.8)	8.9 (6.9 – 11.3)	15.8 (12.3 – 20.1)	0.6 (0.4 – 0.9)
Northeast	During pandemic	1.4 (0.7 – 2.7)	7.3 (4.9 – 10.5)	8.6 (6.0 – 12.1)	81.2 (77.9 – 84.2)	1.4 (0.4 – 4.3)
	In 2023	34.1 (29.7 – 38.8)	34.9 (31.6 – 38.5)	10.1 (7.3 – 13.7)	19.5 (15.9 – 23.6)	1.4 (0.4 – 4.3)
North	During pandemic	1.9 (1.1 – 3.0)	6.8 (4.9 – 9.4)	8.5 (7.3 – 9.9)	82.5 (80.1 – 84.6)	0.2 (0.1 – 0.5)
	In 2023	42.9 (37.5 – 48.7)	34.5 (31.2 – 37.9)	7.0 (4.4 – 11.1)	14.8 (12.7 – 17.2)	0.7 (0.4 – 1.3)
Southeast	During pandemic	0.9 (0.5 – 1.8)	7.4 (4.7 – 11.5)	8.9 (6.1 – 12.9)	82.1 (76.6 – 86.5)	0.6 (0.2 – 1.9)
	In 2023	40.0 (37.6 – 49.7)	33.7 (28.5 – 39.3)	8.7 (6.2 – 12.2)	16.9 (12.4 – 22.6)	0.7 (0.3 – 0.9)
South	During pandemic	1.0 (0.6 – 1.8)	6.4 (4.7 – 8.8)	10.2 (7.4 – 13.9)	81.9 (78.4 – 85.0)	0.3 (0.1 – 0.9)
	In 2023	54.1 (46.4 – 61.6)	31.2 (26.4 – 36.5)	5.9 (4.4 – 7.9)	8.4 (6.2 – 11.3)	0.4 (0.2 – 0.9)
Sex						
Male	During pandemic	1.8 (1.2 – 2.9)	10.3 (7.8 – 13.5)	13.8 (11.0 – 17.2)	73.1 (68.8 – 77.1)	0.9 (0.3 – 2.9)
	In 2023	53.6 (48.2 – 58.9)	28.3 (24.8 – 32.1)	6.9 (5.3 – 9.1)	10.3 (8.3 – 12.7)	0.9 (0.3 – 2.9)
Female	During pandemic	0.6 (0.4 – 1.1)	4.0 (2.6 – 6.2)	4.6 (3.3 – 6.4)	90.1 (87.4 – 92.4)	0.7 (0.3 – 1.3)
	In 2023	29.3 (23.6 – 35.8)	38.4 (35.1 – 41.9)	10.1 (7.9 – 12.8)	21.5 (17.8 – 25.6)	0.8 (0.5 – 1.4)
Education						
0‒8 years	During pandemic	1.0 (0.6 – 1.7)	7.9 (5.3 – 11.9)	7.8 (5.5 – 10.9)	82.2 (77.9 – 85.9)	0.9 (0.4 – 2.0)
	In 2023	36.5 (29.7 – 43.9)	33.3 (29.8 – 36.9)	7.4 (5.8 – 9.3)	21.8 (17.3 – 27.1)	1.1 (0.5 – 2.0)
9‒11 years	During pandemic	1.4 (0.4 – 2.4)	7.7 (5.3 – 11.1)	10.6 (7.6 – 14.6)	79.5 (74.9 – 83.4)	0.8 (0.2 – 4.5)
	In 2023	45.2 (39.3 – 51.2)	32.5 (28.1 – 37.3)	9.4 (7.1 – 12.5)	12.1 (9.3 – 15.7)	0.8 (0.1 – 4.6)
12+ years	During pandemic	1.4 (0.9 – 2.2)	4.1 (3.3 – 5.2)	9.3 (8.3 – 10.4)	84.7 (82.6 – 86.6)	0.5 (0.3 – 0.9)
	In 2023	44.2 (38.3 – 50.4)	35.5 (32.3 – 38.9)	9.8 (7.7 – 12.4)	9.9 (8.1 – 12.2)	0.5 (0.3 – 0.8)
Skin color						
White	During pandemic	1.3 (0.7 – 2.1)	7.8 (5.6 – 10.8)	9.9 (7.2 – 13.6)	80.8 (76.4 – 84.6)	0.2 (0.1 – 0.3)
	In 2023	44.2 (35.8 – 52.9)	33.2 (29.6 – 37.1)	7.9 (5.9 – 10.7)	14.3 (10.8 – 18.7)	0.3 (0.2 – 0.5)
Non-white	During pandemic	1.2 (0.8 – 1.9)	6.6 (4.9 – 7.6)	8.2 (6.8 – 9.9)	83.3 (80.2 – 85.9)	0.7 (0.4 – 2.5)
	In 2023	38.2 (33.9 – 42.6)	34.0 (31.1 – 37.1)	9.3 (7.5 – 11.4)	17.8 (15.2 – 20.7)	0.7 (0.2 – 2.6)

## Discussion

The Covitel findings highlight the pressing issues surrounding the COVID-19 pandemic in Brazil. The study found no significant difference in vaccination rate or face mask usage based on scholarly levels or skin colors. However, there were significantly more confirmed cases of COVID-19 among people with higher education and white skin color. Additionally, Brazilians with lower education had an almost double hospitalization rate. Since color of the skin and the scholarly levels are a proxy of socioeconomic status in Brazil, these findings suggest inequalities in access to virus testing and, consequently, underreporting of the disease among the lower socioeconomic strata of the population.

The inequalities in access to diagnostic for COVID-19 in Brazil may be due to the structure of the country's public health care system. Since the 1980s, Brazil has provided universal healthcare coverage to its citizens, funded by the federal government and local municipalities. However, this system is insufficiently funded and serves a large population, leading to long waiting times. As a result, over one-fourth of Brazilians chose to invest in private insurance. The public health system was the sole and relatively effective provider of COVID-19 vaccines during the pandemic. The diagnostic tests, however, were insufficiently provided by the public health system and readily available in the private health care system. In 2020, Brazil ranked 94th among the countries that performed the most diagnostic tests per million inhabitants (30,000 tests/million inhabitants).^10^ The Covitel findings suggest that individuals with better financial means may have had easier access to testing than those in disadvantaged subgroups. Noteworthy, the EPICOVID Brazil study, based on the prevalence of serological samples, found that people in disadvantaged groups were more likely to be infected than those from more advantaged social groups,^11^ supporting this hypothesis. These findings also raise the suspicion that the number of COVID-19 cases in Brazil may be even greater than reported. Another study in 2020 already identified a positive association between higher per capita income and diagnosis of COVID-19 and an association between cases of severe acute respiratory infection and lower per capita income.^7^

In addition to the failure in the widespread use of effective diagnostic testing, the compliance with the use of face masks in the first trimester of 2023 revealed inequalities among regions of Brazil, with higher adherence in the Northeast and a lower in the South region, potentially linked to political issues.^12^ A plausible explanation may be related to the inequalities that exist between Brazilian regions, with greater resource generation in the southeast and south regions.^13^ The use of face masks and the complete COVID-19 vaccination scheme, also lower among people with higher scholarly, may also be linked to political identification.^14^

Six months after the COVID-19 diagnosis, almost half the infected persons reported decreased memory and a third reported fatigue or low muscle strength amid a myriad of other debilitating complaints potentially related to the infectious disease. Similar findings have been described in Europe^15^ and the US.^16^ An observational study conducted in Italy found that 87 % of the patients discharged from hospital after acute COVID-19 reported symptoms like fatigue (53 %) and joint pain (27 %). A prospective cohort conducted in Wuhan, China, evaluated 1733 patients six months after the COVID-19 acute infection. They found that 76 % of the patients presented at least one symptom, with fatigue and muscular weakness being the most reported.^17^ A survey conducted in the US on 14,767 patients at least two months after a test-confirmed COVID-19 infection found that 1683 individuals presented persistent symptoms. Most of those (57 %) reported at least one cognitive symptom, which was associated with lower functioning, lower full-time employment, and depressive symptoms.^18^

Some limitations in this research must be considered when interpreting the results: a) Survey respondents may suffer from recall bias; b) The greater coverage of telephone lines in capital and metropolitan regions may cause selection bias; c) Bias arising from the profile of the interviewee, as those who answer the phone may have a different profile from those who refuse to participate in the survey; and d) The present study did not differentiate between the types of vaccines administered to each individual, and some vaccines were single-dose. However, single-dose vaccines were administered to a small percentage of people in Brazil. In addition to the inherent limitations of the Covitel study, the diagnosis of COVID-19 was based on laboratory testing and self-reported. Consequently, individuals who had COVID-19 but were not tested did not respond to questions regarding hospitalization and long COVID-19. The strongest point of the present study is its design, as it is the first Brazilian nationwide survey to include mobile phone calls. Considering that 89 % of the urban population and 71 % of rural areas in Brazil use mobile phones,^19^ including mobile phones in the sample is essential to attaining the entire population's representativeness.

In conclusion, the survey revealed almost twice the COVID-19-associated hospitalization rate among people with less education, despite a lower occurrence of confirmed COVID-19, higher use of face masks, and no reported disadvantage in vaccination rate in the lower education strata. These findings suggest inequalities in COVID-19 testing in Brazil, leading to inaccuracies in the epidemiological profile of the pandemic in this country.

## Conflicts of interest

The authors declare no conflicts of interest.
